# Validation of G6PD Point-of-Care Tests among Healthy Volunteers in Yangon, Myanmar

**DOI:** 10.1371/journal.pone.0152304

**Published:** 2016-04-01

**Authors:** Nwe Nwe Oo, Germana Bancone, Lwin Zar Maw, Nongnud Chowwiwat, Pooja Bansil, Gonzalo J. Domingo, Moh Moh Htun, Kyaw Zin Thant, Ye Htut, Francois Nosten

**Affiliations:** 1 Department of Medical Research (Lower Myanmar), Yangon, Republic of the Union of Myanmar; 2 Shoklo Malaria Research Unit, Mahidol–Oxford Tropical Medicine Research Unit, Faculty of Tropical Medicine, Mahidol University, Mae Sot, Thailand; 3 Diagnostics Program, PATH, Seattle, WA, United States of America; 4 Centre for Tropical Medicine, Nuffield Department of Medicine, University of Oxford, Oxford, United Kingdom; Agency for Science, Technology and Research—Singapore Immunology Network, SINGAPORE

## Abstract

Primaquine and other 8-amnoquinoline based anti-malarials can cause haemolysis in subjects with glucose-6-phosphate dehydrogenase (G6PD) deficiency. Correct diagnosis of G6PD status in patients is crucial for safe treatment of both relapsing stages of *Plasmodium vivax* and transmitting forms of *Plasmodium falciparum*. Lack of suitable point-of-care tests has hampered a much needed wide use of primaquine for malaria elimination. In this study we have assessed the performances of two qualitative tests, the fluorescent spot test (FST) and the G6PD CareStart test (CST), against the gold standard quantitative spectrophotometric assay in a population of 1000 random adult healthy volunteers living in Yangon, Myanmar. The prevalence of G6PD deficiency in the Bamar, Karen and in the whole sample set was 6.6% (10.1% in males), 9.2% (21.0% in males) and 6.8% (11.1% in males) respectively. The FST and CST showed comparable performances with sensitivity over 95% and specificity over 90%, however for cases with severe G6PD activity the FTS had improved performance. If used with a conservative interpretation of the signal, the CareStart test has the potential to be used in the field and, by allowing a wider use of primaquine, to help malaria elimination.

## Introduction

Glucose-6-Phosphate Dehydrogenase (G6PD) is the enzyme responsible for maintaining redox equilibrium in red blood cells (RBC). Mutations on the gene result in a decrease of the enzyme stability and activity which translates in increased risk of haemolysis under oxidative challenge at the cell level. The G6PD gene is on the X-chromosome such that males are either G6PD deficient or normal both in terms of genotype and phenotype whereas females with two alleles can be homozygous normal or deficient, but heterozygous females with a deficient G6PD allele and a normal G6PD allele show a range of activities from a completely normal to a completely deficient phenotype depending on their specific pattern of X-inactivation [1]. More than 150 genetic variants have been described worldwide [2], a number of which had a quantitative phenotypic characterization too. In Southeast Asia the two major mutations are Viangchan (found in Thailand, Vietnam, Laos, Cambodia; [3–6]) and Mahidol (Myanmar, Thailand; [7]). Both Viangchan and Mahidol are associated to very low residual enzymatic activity in hemizygous males [8, 9].

G6PD deficiency was first discovered in subjects treated with pamaquine and primaquine (antimalarial 8-aminoquinolines) who experienced a clear drug dose-dependent decrease in hemoglobin concentration after treatment and were therefore defined as being “primaquine” sensitive [10]. Primaquine, together with other 8-aminiquinoline and sulpha drugs, can trigger haemolysis in subjects with G6PD deficiency. Primaquine is the only licensed drug for radical cure of *Plasmodium vivax* malaria. Radical cure of liver dormant stages of *P*. *vivax* is achieved with a 14 day regimen in G6PD normal patients or an eight weeks treatment with a weekly dose in G6PD deficient patients. The WHO recommends G6PD testing before using primaquine for the radical cure of *P*.*vivax* [11]. Diagnosis of G6PD deficiency can be cumbersome. Laboratory based tests, including spectrophotometry [12] and cytochemical staining [13], are expensive, often laborious and require well equipped laboratories and trained technicians. The fluorescent spot test (FST) [14] is probably the most used qualitative screening test for G6PD but requires a cold chain for reagents and electricity for test reading. Rapid diagnostic tests have only recently become available, the CareStart (CST) test, a visual dye colorization test developed by AccessBio, is a promising “point-of-care” test with good sensitivity and specificity [15–17].

In this study we have assessed the performances of two available G6PD qualitative tests: the FST and the CST compared to the reference gold standard spectrophotometric assay, in Myanmar.

## Materials and Methods

Between December 2014 and March 2015, 1000 adult healthy volunteers were randomly recruited among persons who were attending the Out Patient Department of Township Medical Centers of Ahlone and Insein Townships in Yangon Region, Myanmar. After signing informed consent, volunteers had a clinical examination and a 2ml venous blood sample was collected in EDTA tubes. Samples were transported refrigerated to the Department of Medical Research (DMR) Biochemistry laboratory and analyzed for complete blood count and G6PD phenotypes within 6 hours.

Malaria RDT (SD Malaria Ag P.f/P.v, Standard Diagnostic, Korea) was performed as per manufacturer instructions using 5μl of whole blood.

The Complete Blood Count (CBC) was performed using a hematology analyzer (pocH-100i, Sysmex, USA). The CBC included white blood cells (WBC) total and 3-part differential count, red blood cells number (RBC), red blood cells size (MCV), haemoglobin content (MCH and MCHC), total haemoglobin concentration (HGB), haematocrit (HCT) and platelets count (PCT). Quality controls were run every day before analysis of samples. Anaemia was defined by Hb ≤11.5g/dL [18].

Hemoglobin typing (Hb typing) was performed using IsoElectric Focusing (IEF) electrophoresis according to protocol from Gianazza and colleagues [19]. The technique allows for detection of abnormal structural hemoglobins (such as HbE, HbC and HbS). Reticulocyte count on 1000 red blood cells was performed using New Methylene Blue staining. Both Hb typing and reticulocyte count were performed at the Pathology laboratory of DMR.

The G6PD spectrophotometric assay (G-6-PDH quantitative kit, code345-B, Trinity Biotech, Ireland) was performed in duplicate using 10μL of whole blood per replicate; instructions from supplier were followed for reagents preparation. A UV spectrophotometer (UV mini-1240, SHIMADZU, Japan) with electronically controlled temperature compartment was used to detect the absorbance at 340 nm during 10 minutes at 30°C. G6PD activity was calculated as IU/gHb and IU/RBC using the results of the complete blood count on the same blood. Normal, intermediate and deficient G6PD controls (code G6888, G5029 and G5888) were run in double at the beginning of each analysis day; their assessed activity was compared to reference activity and concordance between replicates was analyzed in terms of coefficient of variance (CV). Training on the spectrophotometric assay was performed both in SMRU and at DMR Biochemistry laboratory.

The G6PD fluorescent spot test (FST) (code 203-A, Trinity Biotech, Ireland) was performed using 5μL of blood mixed with 100μL kit reagents. After 10 minutes of incubation at room temperature, a 15μL aliquot was spotted on filter paper and allowed to air dry. The spots were then visualized under UV light; spots that showed fluorescence were classified as normal, spots that failed to show fluorescence were classified as deficient. A normal and a deficient control sample were analyzed along with each batch of samples.

The CareStart test (AccessBio, USA) was performed according to the manufacturer’s instructions: 2 μL of blood were placed in the device and the buffer added immediately; after 10 minutes the reading window was inspected for development of color. Tests showing a pink color were classified as normal, tests showing very faint or no color were classified as deficient, tests that showed remaining blood in the reading window were considered invalid and repeated. Remarks about the uniformity of color in the reading window were collected.

Both G6PD qualitative tests were read by two laboratory technicians unaware of the sprectrophotometric results. If the results were discordant a third reading was performed by a third laboratory technician. Although great effort was put in the initial training and in obtaining only a binary result (deficient or normal) in some cases it was not possible to decide either way and an “Intermediate” result was reported.

Laboratory temperature and humidity were recorded every day at the beginning of sample processing. Temperature range was 24.1–27.1°C and humidity range was 41.1–58.1%.

### Statistical analysis

Data were analyzed using SPSS v22 and SStata 13.1 (Statacorp, College Station, TX). Chi-square test was used to compare proportions among groups. The adjusted population median of G6PD activity was calculated according to [20]. G6PD deficiency by spectrophotometer was defined by <30% of normal activity based on the adjusted population median. Sensitivity, specificity, Negative Predictive Value (NPV), Positive predictive value (PPV) of both FST and CST were calculated in the whole population and in females and males separately, with intermediate results included either in the normal or in the deficient group. Since it is not established yet which activity threshold best predicts a high risk of haemolysis under oxidative challenge, three thresholds of activity (20%, 30% and 40% of population median) were used in the analyses of whole population. The McNemar chi-square test was used to compare sensitivities between different tests or groups. Receiver operating characteristic (ROC) curves for CareStart and FST against the Trinity (gold standard) were plotted and compared; Bonferroni correction was employed to control for multiple comparisons and a p value of <0.05 was used to determine significance (S1 Table). Furthermore, area under the curve (AUC) was plotted against thresholds of G6PD activity for a summarized comparison of the two qualitative tests.

The Oxford University Ethics Committee (ref 22–14) approved the study.

## Results

### Sample and population

A total of 476 women and 524 men were enrolled, mean (SD) age was 39.1 (12.4) years in women and 37.6 (13.6) years in men. The two major ethnic groups were Bamar (76.3%) and Karen (11.9%); less commonly represented were subjects of mix ethnicity (3.2%), Rakhine (2.8%), Chin (1%) and Talgu (0.5%) ethnic groups.

### Blood characterization

None of the study volunteers was found positive for malaria parasites using the RDT.

A summary of hematologic features according to sex is reported in Table 1. With the exclusion of reticulocyte count, all hematologic features were significantly different (P<0.01) between females and males. Anemia was found in 16.2% of women, almost 8 times more than in men (2.5%), OR = 7.59 (95%CI: 4.0–14.5 P<0.001).

**Table 1:** Haematologic characteristic of study population according to gender [Mean (SD)].

	Report									
Sex	N	WBC	RBC	HGB	HCT	MCV	MCH	MCHC	PLT	Reticulocytes
F	476	9.4	5.12	12.6	40.6	78.1	25.0	31.6	311	0.57
		(2.3)	(0.56)	(1.3)	(20.1)	(7.1)	(4.3)	(1.5)	(75)	(0.70)
M	524	8.9	5.71	14.7	45.4	79.9	26.13	32.3	287	0.58
		(2.1)	(0.72)	(1.7)	(4.6)	(7.5)	(4.13)	(1.2)	(80)	(0.91)

Hb typing analysis identified 7 subjects with homozygous hemoglobin E (HbEE), 169 subjects heterozygous for hemoglobin E (HbAE). One subject was diagnosed as HbAD and for two subjects a final diagnosis could not be reached. The remaining 821 had normal Hb type (HbAA) giving an allelic frequency of HbE of 9.2%. Subjects with HbAE and HbEE showed increased RBC number and decreased Hb concentration and smaller RBC size (MCV), Table 2. Anaemia was found in 7.8% of Hb normal subjects, 12.4% of HbAE subjects and 71.4% of HbEE subjects (P<0.001).

**Table 2:** Red Blood cells characteristics [Mean (SD)] according to Hb type.

Hb type		N*	RBC	HGB	HCT	MCV	MCH	MCHC	Reticulocytes
AA	Mean	821	5.36	13.7	43.4	80.3	26.0	32.0	0.59
	SD		(0.67)	(1.8)	(15.7)	(6.6)	(4.4)	(1.5)	(0.82)
AE	Mean	169	5.70	13.6	42.2	74.1	23.8	32.0	0.50
	SD		(0.80)	(1.9)	(5.5)	(7.01)	(2.2)	(0.9)	(0.83)
EE	Mean	7	6.20	11.1	34.8	56.0	17.8	31.9	0.46
	SD		(1.22)	(2.7)	(8.1)	(3.6)	(1.3)	(0.5)	(0.22)

* Three subjects with HbAD and unknown Hb type were not included.

Mean reticulocyte count was not different by Hb type or G6PD phenotype by spectrophotometry.

### G6PD activity by gold standard spectrophometric assay

The adjusted median (range) activity among the normal male population was 8.28 IU/gHb (0.93–18.50) and 213.59 U/RBC (23.79–567.81). Deficiency was found in 6.6% of all Bamar subjects (10.1% in males) and 9.2% of all Karen subjects (21.0% in males)(P_Fisher_ = 0.045). The overall prevalence across the population was 6.8% (11.1% in males).

**Fig 1:**
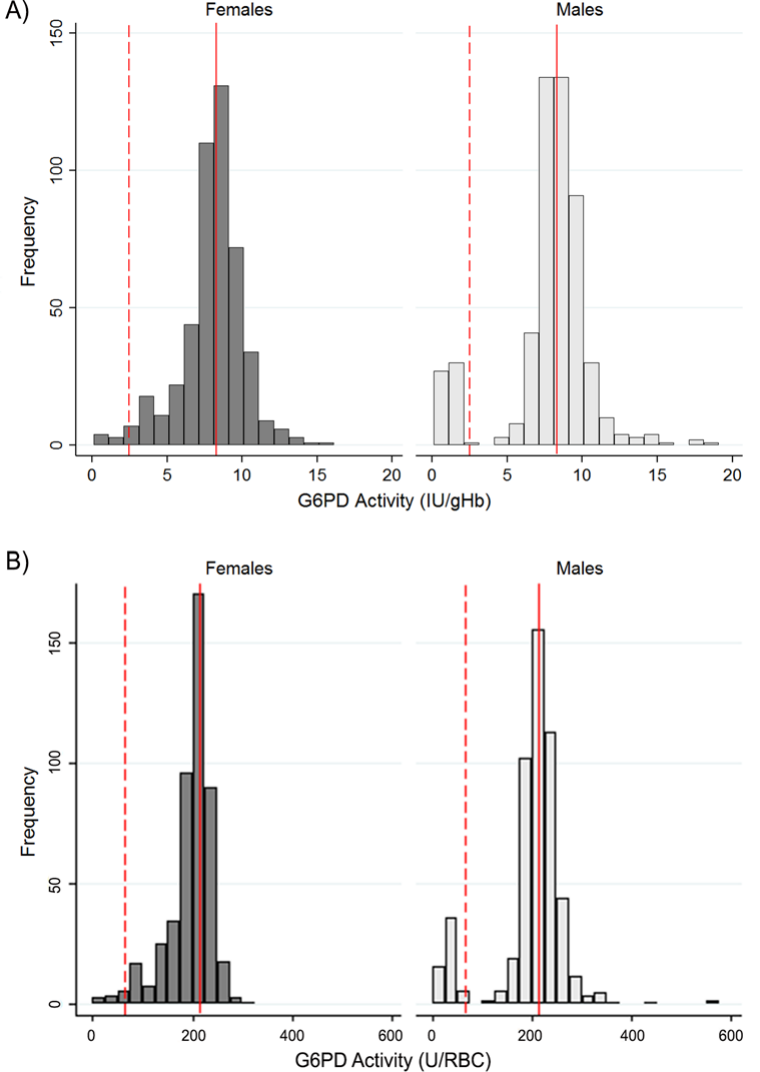
Distribution of G6PD activity in male and female population. A: Histograms for ranges of G6PD activity for both males and females based on G6PD activity normalized for hemoglobin concentration. B: Histograms for ranges of G6PD activity for both males and females based on G6PD activity normalized for red blood cell count. The red vertical reference lines are the adjusted population median, the red dashed reference lines are the 30% activity threshold.

Fig 1 shows the distribution of enzymatic activity by sex. Female subjects showed a single distribution while males showed a clear bi-modal distribution. When enzymatic activity was expressed as IU/gHb the distributions were larger around the population median as compared to the enzymatic activity expressed as U/RBC.

**Fig 2:**
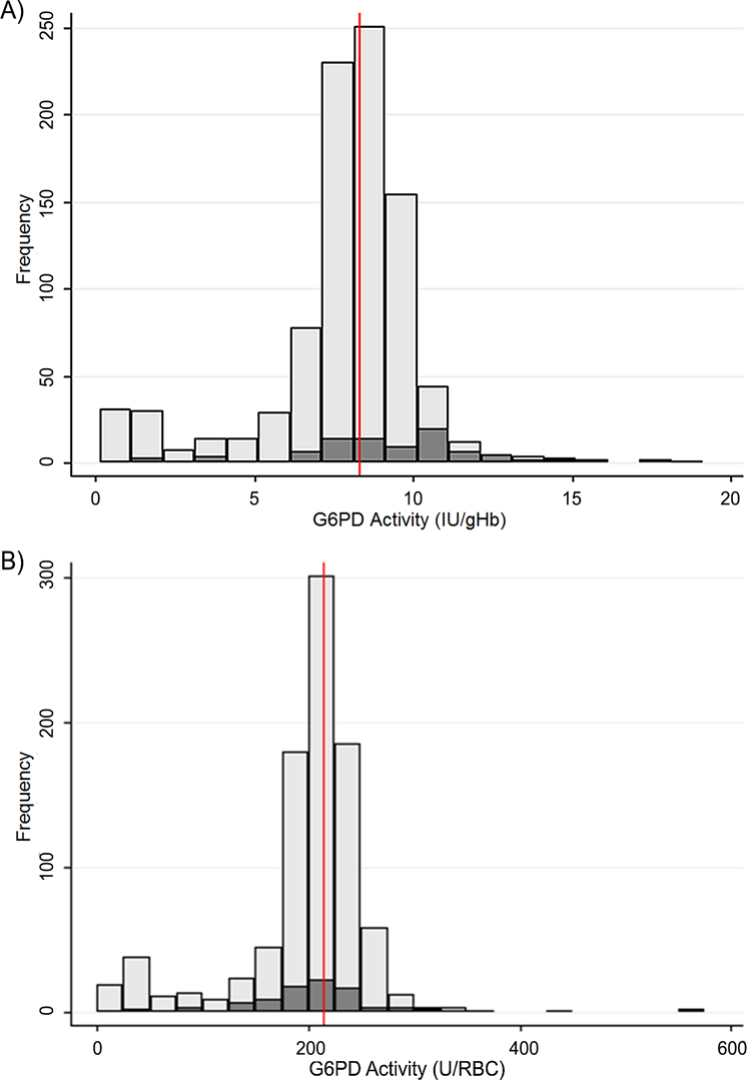
**Distribution of G6PD activity in women with or without anemia shown as histograms for G6PD activity normalized for (A) hemoglobin concentration and (B) red blood cell count (RBC).** Vertical reference line is the population median.

Distribution of activities in anaemic and non-anaemic women is shown in Fig 2A and 2B. In Fig 2A, where activity is expressed as IU/gHb, anaemic women showed a skewed distribution to the right while in Fig 2B, where activity is expressed as U/RBCs, anaemic and non-anaemic women showed the same distribution around the population median. This gives an indication that, especially in anaemic subjects, expressing the enzymatic activity per number of RBCs rather than per grams of hemoglobin might be more accurate.

### Performances of qualitative tests FST and CST

**Fig 3:**
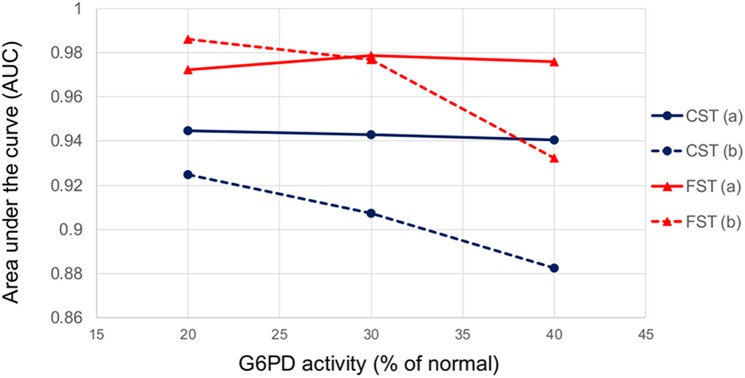
Area under the curve (AUC) vs threshold of G6PD activity (IU/gHb) in the two qualitative tests. FST results with (a) intermediates combined with deficient test results in red solid lines, (b) intermediates combined with normal test results in red dashed lines. CST results with (a) intermediates combined with deficient test results in blue solid lines, (b) intermediates combined with normal test results in blue dashed lines.

Tables 3 and 4 present the data of clinical performances of FST and CST at three different enzymatic activity thresholds (20%, 30% and 40%) using activity expressed either as IU/gHb or as U/RBC. The analysis was performed by including the intermediate results either in the deficient or in the normal group. For the CST, the sensitivity at each threshold was generally higher when intermediate results were pooled with deficient; for the FST sensitivity was the same at the 20% threshold and was slightly higher when intermediate were pooled with deficient at the other two thresholds. Area under the curve analysis suggested that severe G6PD deficient cases are more consistently correctly diagnosed by the fluorescent spot test regardless of whether intermediate spots are interpreted as deficient or normal (S1 Table and Fig 3).

**Table 3:** Clinical performances of the CareStart and Trinity Fluorescent Spot screening tests for detection of deficient G6PD activity (IU/gHb).*

	Trinity 20% Cut-point	Trinity 30% Cut-point	Trinity 40% Cut-point
**Number with deficient G6PD**	58 (52 males)	68 (58 males)	75 (58 males)
**Test Positivity %**			
CareStart Test^a^	14.50		
CareStart Test^b^	7.90		
Trinity Fluorescent Spot Test^a^	9.30		
Trinity Fluorescent Spot Test^b^	6.70		
**Sensitivity % (95%CI)**			
CareStart Test^a^	98.3 (90.8–100.0)	97.1 (89.8–99.6)	96.0 (88.8–99.2)
CareStart Test^b^	87.9 (76.7–95.0)	83.8 (72.9–91.6)	78.7 (67.7–87.3)
Trinity Fluorescent Spot Test^a^	98.3 (90.8–100.0)	98.5 (92.1–100.0)	97.3 (90.7–99.7)
Trinity Fluorescent Spot Test^b^	98.3 (90.8–100.0)	95.6 (87.6–99.1)	86.7 (76.8–93.4)
**Specificity % (95%CI)**			
CareStart Test^a^	90.7 (88.6–92.4)	91.5 (89.5–93.2)	92.1 (90.2–93.8)
CareStart Test^b^	97.0 (95.7–98.0)	97.6 (96.4–98.5)	97.8 (96.7–98.7)
Trinity Fluorescent Spot Test^a^	96.2 (94.7–97.3)	97.2 (95.9–98.2)	97.8 (96.7–98.7)
Trinity Fluorescent Spot Test^b^	98.9 (98.1–99.5)	99.8 (99.2–100.0)	99.8 (99.2–100.0)
**PPV% (95%CI)**			
CareStart Test^a^	39.3 (31.3–47.8)	45.5 (37.2–54.0)	49.7(41.3–58.1)
CareStart Test^b^	64.6 (53.0–75.0)	72.2 (60.9–81.7)	74.7 (63.6–83.8)
Trinity Fluorescent Spot Test^a^	61.3 (50.6–71.2)	72.0 (61.8–80.9)	78.5 (68.8–86.3)
Trinity Fluorescent Spot Test^b^	85.1 (74.3–92.6)	97.0 (89.6–99.6)	97.0 (89.6–99.6)
**NPV% (95%CI)**			
CareStart Test^a^	99.9 (99.4–100.0)	99.8 (99.2–100.0)	99.6 (99.0–99.9)
CareStart Test^b^	99.2 (98.4–99.7)	98.8 (97.9–99.4)	98.3 (97.2–99.0)
Trinity Fluorescent Spot Test^a^	99.9 (99.4–100.0)	99.9 (99.4–100.0)	99.8 (99.2–100.0)
Trinity Fluorescent Spot Test^b^	99.9 (99.4–100.0)	99.7 (99.1–99.9)	98.9 (98.0–99.5)

* Clinical performance estimated using 1000 participants.

^a^ intermediate test results combined with deficient test results.

^b^ intermediate test results combined with normal test results.

Abbreviations: 95%CI, 95% confidence interval; PPV, positive predictive value; NPV, negative predictive value.

**Table 4:** Clinical performance of the CareStart and Trinity Fluorescent Spot screening tests for detection of deficient U/RBC activity.*

	U/RBC 20% Cut-point	U/RBC 30% Cut-point	U/RBC 40% Cut-point
**Number with deficient G6PD**	54	68	75
**Test Positivity %**			
CareStart Test^a^	9.30		
CareStart Test^b^	6.70		
Trinity Fluorescent Spot Test^a^	14.50		
Trinity Fluorescent Spot Test^b^	7.90		
**Sensitivity % (95%CI)**			
CareStart Test^a^	98.1 (90.1–100.0)	97.1 (89.8–99.6)	96.0 (88.8–99.20
CareStart Test^b^	98.1 (90.1–100.0)	94.1 (85.6–98.4)	86.7 (76.8–93.4)
Trinity Fluorescent Spot Test^a^	98.1 (90.1–100.0)	97.1 (89.8–96.6)	96.0 (88.8–99.2)
Trinity Fluorescent Spot Test^b^	87.0 (75.1–94.6)	85.3 (74.6–92.7)	78.7 (67.7–87.3)
**Specificity % (95%CI)**			
CareStart Test^a^	95.8 (94.3–97.0)	97.1 (95.8–98.1)	97.7 (96.6–98.6)
CareStart Test^b^	98.5 (97.5–99.2)	99.7 (99.1–99.9)	99.8 (99.2–100.0)
Trinity Fluorescent Spot Test^a^	90.3 (88.2–92.1)	91.5 (89.5–93.2)	96.0 (88.8–99.2)
Trinity Fluorescent Spot Test^b^	96.6 (95.3–97.7)	97.7 (96.6–98.6)	78.7 (67.7–87.3)
**PPV% (95%CI)**			
CareStart Test^a^	57.0 (46.3–67.2)	71.0 (60.6–79.9)	77.4 (67.6–85.4)
CareStart Test^b^	79.1 (67.4–88.1)	95.5 (87.5–99.1)	97.0 (89.6–99.6)
Trinity Fluorescent Spot Test^a^	36.6 (28.7–44.9)	45.5 (37.2–54.0)	49.7 (41.3–58.1)
Trinity Fluorescent Spot Test^b^	59.5 (47.9–70.4)	73.4 (62.3–82.7)	74.7 (63.6–83.8)
**NPV% (95%CI)**			
CareStart Test^a^	99.9 (99.4–100.0)	99.8 (99.2–100.0)	99.7 (99.0–99.9)
CareStart Test^b^	99.9 (99.4–100.0)	99.6 (98.9–99.9)	98.9 (98.0–99.5)
Trinity Fluorescent Spot Test^a^	99.9 (99.4–100.0)	99.8 (99.2–100.0)	99.6 (99.0–99.9)
Trinity Fluorescent Spot Test^b^	99.2 (98.4–99.7)	98.9 (98.0–99.5)	98.3 (97.2–99.0)

* Clinical performance estimated using 1000 participants.

^a^ intermediate test results combined with deficient test results.

^b^ intermediate test results combined with normal test results.

Abbreviations: 95%CI, 95% confidence interval; PPV, positive predictive value; NPV, negative predictive value.

Table 5 shows the results of clinical performances of the FST and CST at the 30% threshold for female and male subjects separately. As expected both tests performed better in the male population where the sensitivity was found to be higher and the confidence intervals more narrow compared to females. In males, the FST showed a very high sensitivity (98.3%) regardless of the classification of intermediate results as normal or deficient. The FST showed significantly better performances than CST when intermediate results were pooled with normal (sensitivity 98.3% *vs* 86.2%, P = 0.039).

**Table 5:** Clinical performance of the CareStart and Trinity Fluorescent Spot screening tests for detection of deficient G6PD activity.

	Males (N = 524) 30% Cut-point	Females (N = 476) 30% Cut-point
**Number with deficient G6PD**	58	10
**Test Positivity %**		
CareStart Test^a^	15.84	13.03
CareStart Test^b^	10.88	4.62
Trinity Fluorescent Spot Test^a^	12.60	5.67
Trinity Fluorescent Spot Test^b^	11.07	1.89
**Sensitivity % (95%CI)**		
CareStart Test^a^	98.3 (90.8–100.0)	90.0 (55.5–99.7)
CareStart Test^b^	86.2 (74.6–93.9)	70.0 (34.8–93.3)
Trinity Fluorescent Spot Test^a^	98.3 (90.8–100.0)	100.0 (69.2–100.0)
Trinity Fluorescent Spot Test^b^	98.3 (90.8–100.0)	80.0 (44.4–97.5)
**Specificity % (95%CI)**		
CareStart Test^a^	94.4 (91.9–96.3)	88.6 (85.4–91.4)
CareStart Test^b^	98.5 (96.9–99.4)	96.8 (94.7–98.2)
Trinity Fluorescent Spot Test^a^	98.1 (96.4–99.1)	96.4 (94.2–97.9)
Trinity Fluorescent Spot Test^b^	99.8 (98.8–100.0)	99.8 (98.8–100.0)
**PPV% (95%CI)**		
CareStart Test^a^	68.7 (57.6–78.4)	14.5 (6.86–25.8)
CareStart Test^b^	87.7 (76.3–94.9)	31.8 (13.9–54.9)
Trinity Fluorescent Spot Test^a^	86.4 (75.7–93.6)	37.0 (19.4–57.6)
Trinity Fluorescent Spot Test^b^	98.3 (90.8–100.0)	88.9 (51.8–99.7)
**NPV% (95%CI)**		
CareStart Test^a^	99.8 (98.7–100.0)	99.8 (98.7–100.0)
CareStart Test^b^	98.3 (96.7–99.3)	99.3 (98.1–99.9)
Trinity Fluorescent Spot Test^a^	99.8 (98.8–100.0)	100.0 (99.2–100.0)
Trinity Fluorescent Spot Test^b^	99.8 (98.8–100.0)	99.6 (98.5–99.9)

* Clinical performance estimated using 1000 participants.

^a^ intermediate test results combined with deficient test results.

^b^ intermediate test results combined with normal test results.

Abbreviations: 95%CI, 95% confidence interval; PPV, positive predictive value; NPV, negative predictive value.

### Considerations on the use of the CareStart test kits

**Fig 4:**
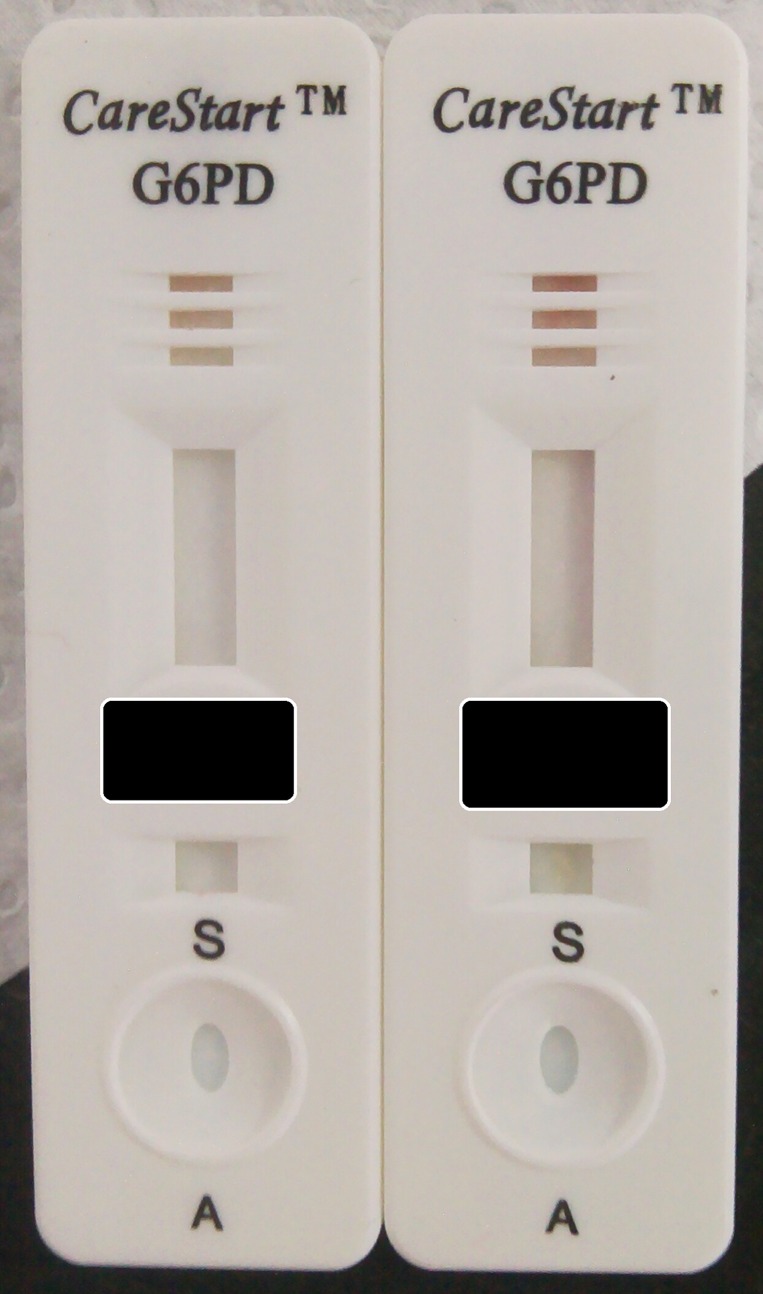
Example of samples from a G6PD deficient male (left, G6PD activity = 1.21 IU/gHb) and G6PD normal male (right, G6PD activity = 7.18 IU/gHb) that were both diagnosed as deficient using the CST test.

The CareStart test comes with a micropipette containing a haematocrit tube for sampling the blood directly from the finger prick. The laboratory technicians found the use of this pipette difficult and transfer of blood from the EDTA tube was performed with a standard micropipette. In 3.4% cases there was still blood in the reading windows and the test had to be repeated. In 4.2% cases the color development was not uniform and in the majority of these cases it developed in the very side of the reading window (Fig 4). In general the color intensity was variable even across normal samples with the same enzymatic activity and this is likely to impact on the final diagnosis; CST classified as “intermediate” twice as much samples as the FST: 66 against 26 in the whole study and 26 against 8 only in males.

## Discussion

### G6PD prevalence and distribution

The overall prevalence of G6PD deficiency in the study was 6.8% in the whole population and 11.1% in males. Previous publications showed a prevalence of G6PD deficiency of 12% in Mon and 10% in Bamar males [7] and 11% in the general Myanmar population of remote areas [21]. Males of Bamar ethnicity at the Thai-Myanmar border were found to have 12.9% prevalence of G6PD deficiency [9]. A study conducted among Bamar subjects of Shan state did not characterize phenotypes but found an allelic frequency for Mahidol deficient mutation of 11.6% [22] (although article reports 21.2% prevalence).

### G6PD activity by spectrophotometry

Distributions of G6PD enzymatic activities in the population are different between males and females, the data obtained in the study showed a clear bi-modal distribution in males and a continuous distribution in females. Based on the prevalence of deficient phenotype in males, roughly corresponding to the frequency of deficient mutation(s), we expect to have in the same population <2% homozygous fully deficient women and 20% heterozygous women with a wide range of activities (overlapping both deficient and normal), Fig 1. The results of enzymatic activity can be expressed in terms of IU/gHb and U/RBC, with the first being the most commonly used. Both normalization by hemoglobin and RBC are influenced by changes in hematologic parameters and in 1967 the WHO [23] specifically recommended using the normalization by RBC in samples with hypochromic RBCs (found in anaemic subjects). Different causes of anaemia might produce different hematologic profiles, together with low Hb levels some subjects might present lower MCV (iron deficiency) and concomitant increased RBC number (typically seen in subjects carriers of abnormal Hb variants and haemoglobinopathies). In the population under study, anaemia caused by several factors is found in over 15% of women and expressing G6PD activity as U/RBC might be more accurate in this portion of population. While anemic subjects with complete G6PD deficiency are expected to be always classified as deficient regardless the normalization used, in women with intermediate G6PD phenotype the normalization of enzymatic activity in terms of IU/gHb might give higher normalized activity results as compared to the enzymatic activity expressed in terms of U/RBCs. This has implications in the treatment decision.

### Validation of qualitative tests

In the present study, both FST and CST were used in laboratory conditions on venous blood collected few hours before. The FST confirmed to be one of the most robust G6PD qualitative tests available, with consistently good performances (sensitivity >95% at 30% or 40% activity threshold) in different laboratory settings [15–17, 24]. Requirement for cold chain and electricity to perform the test makes it a testing option only for laboratory-based or campaign style screenings.

The CST test was found here to be simple to use and interpret and confirmed previous results [15–17, 24] of performances to be similar or slightly inferior to FST (sensitivity >89% at 30% or 40% activity threshold). Results from a recent publication [25] seem to show a very high sensitivity of the test but the threshold used was 75% activity. The number of tests considered invalid because of poor blood migration was also low as seen previously in venous blood; the lack of a control band was considered by the operators a problem, especially when there was no color development in the device as it is the case of deficient samples.

One of the major limitations of qualitative tests in G6PD diagnosis is the reporting of “intermediate” results, which represent a challenge in the assessment of the test performances; since true intermediate activities are found in heterozygous females. In this context reporting intermediate results was allowed. To account for this, performances of the two tests were further compared specifically on samples from male volunteers. In males the FST consistently gave undetectable signal for all males with less than 30% G6PD resulting in a sensitivity of 98.3% regardless of how intermediates were classified, whereas the CST had a sensitivity of 86.2% unless intermediates were classified as deficient in which case the sensitivity increased to 98.3%. Twenty-six male subjects were classified as intermediate by the CST (versus only 8 male classified as intermediate by the FST), an aspect that should be addressed at the manufacturing level probably making the color development more homogeneous within the same device and across devices. Correct diagnosis of G6PD deficiency in females using a qualitative test has intrinsic limitations due to the very biology of G6PD expression; when intermediate results are found in females the operators are faced with a dilemma on how to report the results (especially if they inform treatment decisions). The precautionary approach would suggest reporting a diagnosis of deficiency although this strategy will lead to withhold treatment in some subjects who could actually have been treated safely. With respect to anaemia, the data have shown here that the performances of both qualitative tests are only marginally influenced by Hb levels probably because anemia is more common in females where the tests show lower sensitivity. Quantitative point-of-care tests, which will also include assessment of hemoglobin concentrations, are the only foreseeable tool for reliable assessment of G6PD activity in female patients on the field [11] and will be hopefully available soon.

The present evaluation of the CareStart test, considering both the technical performances and the minimal storage requirements, supports the utility of the test in malaria endemic areas where currently other options are not available. What makes the CST particularly relevant to *P*.*vivax* case management is the point-of-care configuration of the test, therefore the storing and performing conditions (presently 4–30°C) should be adapted for field use in tropical and subtropical countries where ambient temperature might reach 40°C and humidity exceeds 85% (also in line with malaria RDT tests). The data presented here suggest that unless the CST results are interpreted conservatively, some deficient subjects will be classified as normal. Interpretation of the results would be facilitated by incorporation of a control line and more uniform coloring of the reading window. Providing a color chart for helping in interpretation of results would also be of great help. Hopefully field evaluations of the test will confirm in the near future the performances obtained so far in the laboratory settings and G6PD testing and therefore safe radical cure of *P*.*vivax* patients will become available more widely.

## Supporting Information

S1 Table Area under the curve (AUC) for CareStart and FST against the gold standard quantitative spectrophotometry at three thresholds (205, 30% and 40%) of G6PD activity. (XLSX)
